# Immunological Aspects of Fulminant Type 1 Diabetes in Chinese

**DOI:** 10.1155/2016/1858202

**Published:** 2016-02-14

**Authors:** Zhen Wang, Ying Zheng, Yiting Tu, Zhijie Dai, Jian Lin, Zhiguang Zhou

**Affiliations:** ^1^Diabetes Center, 2nd Xiangya Hospital and Institute of Metabolism and Endocrinology, Key Laboratory of Diabetes Immunology, Ministry of Education, Central South University, 139 Renmin Middle Road, Changsha, Hunan 410011, China; ^2^The Center for Medical Research, 2nd Xiangya Hospital, Central South University, 139 Renmin Middle Road, Changsha, Hunan 410011, China

## Abstract

*Background.* Fulminant type 1 diabetes (FT1D) is a novel subtype of type 1 diabetes characterized by extremely rapid onset and complete deficiency of insulin due to the destruction of pancreatic *β* cells. However, the precise mechanisms underlying the etiology of this disease remain unclear.* Methods.* A total of 22 patients with FT1D and 10 healthy subjects were recruited. Serum antibodies to GAD, IA2, and ZnT8 in patients were tested. And peripheral T cell responses to GAD65, insulin B9–23 peptide, or C peptide were determined in 10 FT1D patients and 10 healthy controls. The mRNA levels of several related cytokines and molecules, such as IFN-*γ*, IL-4, RORC, and IL-17 in PBMCs from FT1D patients were analyzed by qRT-PCR.* Result.* We found that a certain proportion of Chinese FT1D patients actually have developed islet-related autoantibodies after onset of the disease. The GAD, insulin, or C peptide-reactive T cells were found in some FT1D patients. We also detected a significant increase for IFN-*γ* expression in FT1D PBMCs as compared with that of healthy controls.* Conclusion.* Autoimmune responses might be involved in the pathogenesis of Chinese FT1D.

## 1. Introduction

Type 1 diabetes is characterized by insulin deficiency resulting from the destruction of pancreatic *β* cells. According to the classification of diabetes by the American Diabetes Association (ADA) and the World Health Organization (WHO), type 1 diabetes is divided into two subtypes: type 1A (autoimmune) and type 1B [1, 2]. Type 1A diabetes is characterized as the abnormal activation of the T cell mediated immune system, leading to an inflammatory response in islets as well as to a humoral response with production of autoantibodies to beta-cell antigens (ICA), insulin (IAA), glutamic acid decarboxylase (GADA), and the protein tyrosine phosphatase IA2 (IA-2A) [3–5]. Recently, the imbalance of T helper (Th) subsets and their secreted cytokines is suggested to be involved in autoimmune inflammation in type 1A diabetes [6]. Th cells have been subdivided into different subsets, including Th1, Th2, Th17, and regulatory T (Treg) cells. Th1 cells predominantly produce Interleukin-2 (IL-2), interferon-*γ* (IFN-*γ*), and tumor necrosis factor-*β* (TNF-*β*), which mediate autoimmune pathology, while Th2 cells mainly release IL-4, and IL-10, leading to a dominant protective effect against autoimmune responses [7]. Th17 cells, which are delineated by their production of proinflammatory cytokines IL-17, is a contributing factor in the onset and progress of type 1A diabetes [8]. Treg cells, which have important regulatory functions in vitro and vivo, play a vital role in controlling effector T cell responses and maintaining immune tolerance [9].

Fulminant type 1 diabetes (FT1D) is a novel subtype of type 1 diabetes first proposed by Japanese scholars in 2000. It is an extremely aggressive disease characterized by the abrupt onset of insulin-deficient hyperglycemia, rapid progression to ketosis or ketoacidosis within a short period, and complete *β*-cell destruction [10]. The precise mechanisms underlying the etiology of this disease almost remain unclear. Initial reports emphasized the absence of autoantibodies and autoimmune responses in FT1D, so that it was divided into the type 1B diabetes. However, subsequent studies showed that autoimmune response is implied in the pathoetiology of FT1D. A national survey performed in Japan revealed that ~5% of FT1D patients have GAD autoantibodies [11]. In 2000, a case report from Tanaka et al. showed insulitis in one FT1D patient's pancreas accompanied with CD8+ T cells infiltration [12]. Furthermore, massive T cells-infiltration in the pancreas has been detected just after disease onset and increased T cell responses against pancreatic *β*-cell antigens were also detected by enzyme-linked immunospot (ELISPOT) assay [13]. In addition, CXCL10 secreted from *β*-cells activates and attracts autoreactive T cells and macrophages to the islets via CXCR3, and the infiltrating autoreactive T cells and macrophages release inflammatory cytokines, thus activating cell-mediated autoimmunity in islet and accelerating *β*-cell destruction in FT1D [14]. Recently, low CTLA-4 expression was found in CD4+ helper T cells from Japanese FT1D patients, which implied a possibility of Treg deficiency in the disease [15]. These observed results were mainly from studies in Japanese and Korean populations.

By studies of FT1D patients in Chinese population, we showed that FT1D also exhibits some distinct clinical and autoimmunity features from classical type 1 diabetes in Chinese patients [16]. We found that Foxp3 and CTLA-4 expressions are reduced in PBMCs from FT1D patients and that deficient Tregs contribute to the pathology of FT1D [17]. To better understand the pathoetiology of FT1D, here we described the autoimmunity features of FT1D from the perspective of humoral and cellular immunity. A total of 22 Chinese patients with FT1D were recruited, and their serum autoantibodies to GAD, IA2, and ZnT8 were measured with radio ligand assay. For 10 of them, GAD, insulin, and C peptide-reactive T cells were measured by ELISPOT assay. In addition, key effectors or regulators of Th cells, including IFN-*γ*, IL-4, RORC, and IL-17, were determined by qRT-PCR. Our findings suggest that a certain proportion of patients with FT1D exhibit autoimmunity features and that autoimmunity is involved in the development of FT1D.

## 2. Subjects Methods

### 2.1. Subjects

We studied 22 patients with FT1D hospitalized into Institute of Metabolism and Endocrinology, 2nd Xiangya Hospital of Central South University between January 2000 and December 2011, diagnosed according to the following inclusion criteria proposed by the Committee of the Japan Diabetes Society: (1) occurrence of diabetic ketosis or ketoacidosis soon (within 7 days) after the onset of hyperglycemic symptoms (elevation of urinary and/or serum ketone bodies at first visit); (2) plasma glucose level >16.0 mmol/L (>288 mg/dL) and glycated hemoglobin (HbA1c) level <8.5% at first visit; (3) urinary C peptide excretion <10 mg/day or fasting serum C peptide level <0.3 ng/mL (0.1 nmol/L) and <0.5 ng/mL (<0.17 nmol/L) after intravenous glucagon load (or after a meal) at onset. 10 age- and sex-matched healthy subjects were served as controls. The study was approved by the human ethics committee of Central South University. Informed consent was obtained from all subjects recruited.

### 2.2. Serum Antibodies Measurement

Serum antibodies to GAD, IA2, and ZnT8 were measured by radio binding assay with in vitro translated 35S-methionine-labelled GAD65, IA-2, or ZnT8. And the results converted into arbitrary units by extrapolation from a standard curve with a local standard. The inter- and intra-assay CV of the serum samples detected by this assay for GADA are 7.1%~10.8% and CV% 4.9%~8.3%; the inter- and intra-assay CV for IA-2A are 5.6%~11.7% and 3.2%~9.7%; and the inter- and intra-assay CV for ZnT8A are 4.3%~13.8% and 3.9%~9.8%. The thresholds of positivity for GADA, IA2A, and ZnT8A were ≥0.05, ≥0.007, and ≥0.011, respectively.

### 2.3. ELISPOT Assay

The ELISPOT assay was performed as previously described. In brief, nitro-cellulose-bottomed 96-well microtiter plates were coated with anti-IFN-*γ* antibody (mAb) (1-DIK, Mabtech AB, Stockholm, Sweden) overnight at 4°C. Unbound antibodies were removed by washing with sterile PBS. Peripheral blood mononuclear cells (PBMCs) were isolated by density gradient centrifugation using lymphocyte separation medium. Aliquots of 2 × 10^5^ PBMCs per well were incubated in mAb-coated plates together with 1 unit/mL interleukin-2 (R&D Systems, Lille, France), and 10 mg/mL GAD65 and 10 mg/mL insulin B9–23 or C peptide, or with phytohemagglutinin as positive control and dimethyl sulfoxide (diluent) as negative control in immunoglobulin-free media for 40 h. Then, biotinylated IFN-*γ*-detection antibody (U-CyTech) in PBS with 10% FCS was added and incubated for 1 h at 37°C, followed by incubation of streptavidin conjugated with alkaline phosphatase for another 1 h. After extensive washings, 5-bromo-4-chloro-3-indolyl phosphate/nitro blue tetrazolium substrate (Sigma-Aldrich) was added to visualize spots. The reaction was stopped 10 min later by washing with water, and then spot counting was performed by using an ELISPOT reader. The cutoff value for GAD65, insulin B9–23 peptide or C peptide-stimulated IFN-*γ* positivity was set at the mean + 3 standard deviation of the healthy control population.

### 2.4. RNA Isolation and Real-Time Quantitative PCR

Total PBMC RNA was isolated using the Trizol reagents as instructed (Invitrogen, San Diego, CA, USA). cDNA was synthesized from 1 *μ*g of total RNA using a reverse transcription kit from Promega (Madison, WI, USA). Real-time quantitative PCR was conducted using IQ5 (BioRad, Hercules, CA, USA). The experiments were performed with 20 *μ*L reaction volumes containing 10 *μ*L 2x SYBR® Premix Ex Taq (TaKaRa Biotech, Dalian, China), 0.4 *μ*M of each primer, 1 *μ*L of cDNA template, and 8.2 *μ*L deionized water. PCR amplifications were done using the following parameters: 95°C for 15 s, 40 cycles through 95°C for 5 s, 60°C for 30 s. Melting curve analyses were also performed to exclude nonspecific PCR products. For each biological sample, technical triplicates were made. mRNA levels were determined by real-time PCR using the primers as follows: IFN-*γ* forward, GTGGAGACCATCAAG GAAGAC; IFN-*γ* reverse, TATTGCTTTGCGTTGGACAT; IL-4 forward, CGGCAGTTC TACAGCCACCA; IL-4 reverse, TCCTGTCGAGCCGTTTCAG; RORC forward, TGAGAAGGACAGGGAGCCAA; RORC reverse, CCACAGA TTTTGCAAGGGATCA; IL-17 forward, GCTACGGTGCAG GTAAAGTTC; IL-17 reverse, GCAGAAGTGCATTTGAC AAGAGA; GAPDH forward, ATCAAGATCAT TGCTCCTCCTGAG; GAPDH reverse, CTG CTTGCTGATCCACATCTG.

### 2.5. Statistical Analysis

All statistical analyses were conducted using the SPSS 13.0 software (Chicago, IL, USA). Results are expressed as mean ± SD. Data were analyzed by ANOVA followed by the Student's *t*-test. In all cases, *p* < 0.05 was considered with statistical significance.

## 3. Results

### 3.1. Evaluation of Serum Autoantibodies in FT1D

All 22 of FT1D patients participating in this study displayed rapid diabetic ketoacidosis with little insulin secretion (F-CPR < 0.07 mM).  Table 1 shows the basic clinical characteristics of FT1D patients in this study. Analysis of serum autoantibodies revealed that 9 out of 22 cases exhibited lower titres of autoantibodies against one or more autoantigens, including 5 cases positive for GADA, 2 for IA-2A, 3 for ZnT8A. Particularly, one case showed both GADA and ZnT8A (Table 2).

### 3.2. Detection of *β*-Cell Specific-Reactive T Cells in FT1D

**Figure 1:**
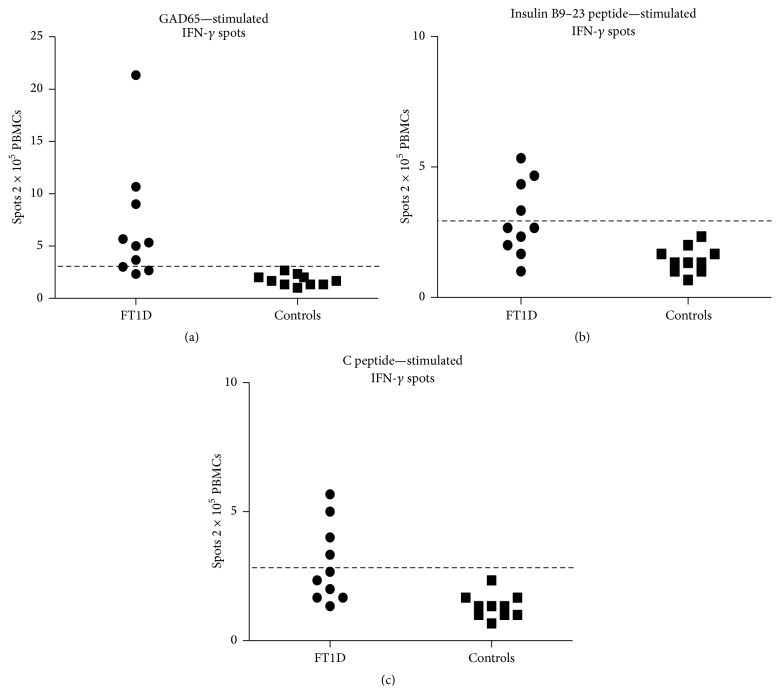
Comparison for the IFN-*γ* secretion induced by GAD65 and insulin B9–23 and C peptide. The average spot of all individual IFN-*γ* spots are depicted as a separate marker, control subjects (closed squares), and FT1D patients (closed circles). Antigen-stimulated spots were determined as follows: [(the mean number of spots in the presence of antigen) − (the mean obtained without stimulation)]. Each horizontal line represents the mean + 3 SD of the healthy controls.

To determine whether *β*-cell specific-reactive T cell autoimmunity is participated in the pathogenesis of FT1D, we used ELISPOT assays to detect T cell response to GAD65, insulin B9–23 peptide, or C peptide in 10 FT1D patients, and 10 healthy controls. We used phytohemagglutinin as positive control and dimethyl sulfoxide (diluent) as negative control. As expected, there was no GAD65-specific T cells in healthy controls in this ELISPOT assay. The cutoff value for GAD65, insulin B9–23 peptide, or C peptide-stimulated IFN-*γ* positivity was set at the mean + 3 standard deviation of the healthy control population. In FT1D patients, IFN-*γ* spots in response to GAD and insulin B9–23 and C peptide were significantly detected in 70%, 40% and 40%, respectively (Figure 1, Tables 3 and 4).

### 3.3. Determination of Cytokines for Th1/Th2 and Th17 Cells

**Figure 2:**
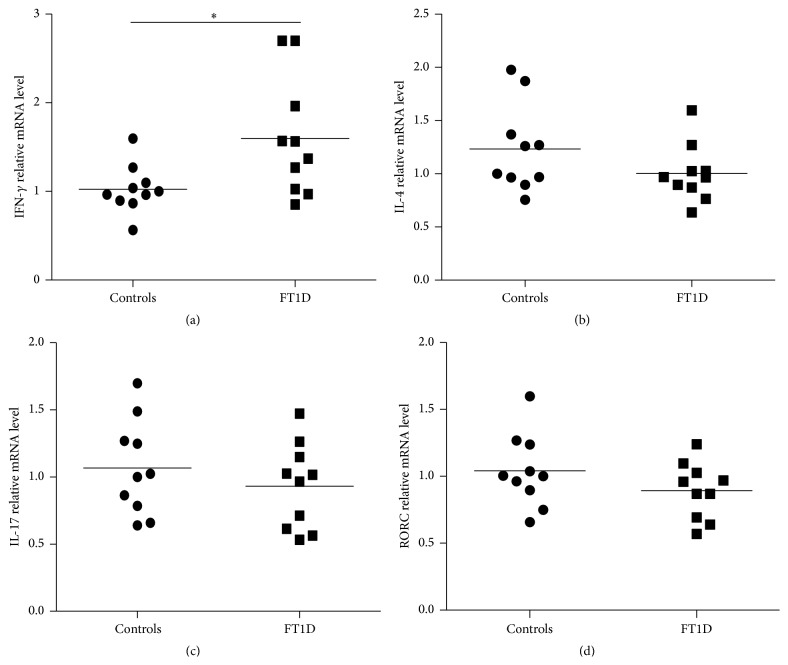
IFN-*γ* expression levels are increased in PBMCs from patients with FT1D. Relative mRNA levels for IFN-*γ*, IL-4, IL-17, and RORC in PBMCs from FT1D and healthy controls (*n* = 10). The expression levels were normalized by GAPDH. Data plotted represent expression levels in PBMCs of control subjects (closed circles) and FT1D patients (closed squares).

To further evaluate whether FT1D exerts aberrant Th cell immunity, we determined the mRNA levels of several related cytokines and molecules, such as IFN-*γ*, IL-4, RORC, and IL-17 in PBMCs from FT1D patients. We observed a significant increase for IFN-*γ* expression as compared with that of healthy controls. Unexpectedly, we failed to detect a significance alteration for IL-4, RORC, and IL-17 expression between all patients and controls (Figure 2).

## 4. Discussion

Previous reports suggested that autoimmunity is not involved in FT1D because of no detection for diabetes-related autoantibodies [10]. However, the followup large-scale studies by Japanese scholars revealed that a proportion of FT1D patients actually have developed islet-related autoantibodies, such as GAD5 [11]. At present, the single detection for GADA is still the only critical determinant of autoimmunity in FT1D patients in Japan. However, the combined detection of GADA, IA-2A, and ZnT8A was applied in our study, which would promote the efficiency of diagnosis for autoimmunity in FT1D patients. In our study, we determined serum autoantibodies against GAD, IA-2, and ZnT8 in 22 Chinese patients with FT1D after the onset of diabetes, and we found 5 cases positive for GADA, 2 for IA-2A, and 3 for ZnT8A. And one of 22 cases showed both GADA and ZnT8A.

Recent studies have shown that T cell mediated autoimmunity is involved in FT1D [13]. ELISPOT assay has been used to detect T cell reactivity. By ELISPOT assay, 66.7% of Type 1 diabetes patients were found to have T cell reactivity to islet cell antigens [18]. Kotani et al. reported that nine of 13 (69.2%) GAD-reactive T cells and three of 12 (25%) insulin-B9–23-reactive T cells were identified in Japanese FT1D patients by ELISPOT [13]. In this study, we detected the T cell reactivity to GAD65, insulin B9–23 peptide, or C peptide in 10 Chinese FT1D patients by ELISPOT assays. The GAD-stimulated IFN-*γ* spots were detected in 7 FT1D patients, and both insulin and C peptide-stimulated IFN-*γ* spots were detected in 4 FT1D patients, and different antigen-stimulated IFN-*γ* spots were also detected in the same case. T cell reactivity is mainly mediated by the GAD antigen in FT1D, and the detection of C peptide or insulin-stimulated IFN-*γ* spots could help us to understand immunological features of FT1D. These results suggest that autoreactive T cells might contribute, at least in part, to the development of Chinese FT1D.

The relative pathogenic effects of Th1 cells and protective contributions of Th2 cells have been recognized as a common feature of T1D. Th1 populations, which secrete cytokines, including IFN-*γ* and IL-2, can regulate *β*-cell autoreactivity, while Th2 populations, which are associated with the secretion of cytokines, such as IL-4, IL-5, and IL-13, result in a dominant protective effect against T1D [6]. Recent studies have shown that Th17 cells are involved in the pathology of T1D, which produce the inflammatory cytokines IL-17, IL-22, and IL-21 [8]. Among these cytokines, IL-17 is capable of inducing the synthesis of other cytokines and chemokines, resulting in autoimmune responses [19]. Meanwhile, Th17 cells are regulated by some cytokines and transcription factors [20]. Among them, transcriptional factor RAR-related orphan receptor gamma (RORC) is required for the development of the cells [21]. Here, we determined the mRNA expressions of IFN-*γ*, IL-4, IL-17, and RORC in the PBMCs from 10 FT1D patients and found a significant increase for IFN-*γ* expression in FT1D PBMCs as compared with that of healthy controls. The result suggested that abnormal Th1-cell immunity seems to contribute to the pathogenesis of FT1D. Although we failed to detect the difference in the expression of IL-4, IL-17, and RORC between the FT1D patients and healthy controls, we cannot conclude that Th2-cell immunity or Th17-cell immunity is not involved in the development of FT1D because of the limitation of determining the expressions of these molecules in PBMCs. Therefore, further studies are needed to confirm these questions.

Increasing studies showed that autoimmunity is considered to be a cause of the markedly rapid loss of *β* cells in FT1D. A better understanding underlying FT1D pathoetiology will provide us with new markers for an early diagnosis of the disease and strategies for prevention or treatment of this devastating disorder. Our previous study demonstrated that Treg deficiency contribute to the development of FT1D. In this study, our results show that a low proportion of Chinese FT1D patients actually have developed islet-related autoantibodies after onset of the disease and demonstrate that autoreactive T cell immune responses might be involved in the pathogenesis of Chinese FT1D. The study also suggested that Th1-cell immune responses might be participated in the development of FT1D. Although our data provide some evidence that autoimmunity is involved in the onset and development of FT1D, further detailed studies would be necessary to clarify the mechanisms of FT1D pathogenesis.

## Figures and Tables

**Table 1:** Basic clinical characteristics of FT1D patients in this study.

	The whole subjects (*N* = 22)
Ages (years)	27.8 ± 7.6
Gender (male), *n* (%)	8 (36.4)
Duration of diabetes (days)	2.7 ± 1.6
Family history of diabetes, *n* (%)	1 (4.5)
Flu-like symptoms, *n* (%)	6 (27.2)
Gastrointestinal symptoms, *n* (%)	14 (63.6)
Disturbance of consciousness, *n* (%)	3 (13.6)
Body mass index (kg/m^2^)	20.2 ± 3.4
Plasma glucose (mM)	40.2 ± 15.9

**Table 2:** Distribution of GADA, IA-2A, and ZnT8A in 22 Chinese FT1D patients.

Serum antibody	FT1DM (*n* = 22)
GADA	5/22
IA-2A	2/22
ZnT8A	3/22
GADA and IA-2A	0/22
GADA and ZnT8A	1/22
IA-2A and ZnT8A	0/22
GADA, IA-2A, and ZnT8A	0/22
GADA, IA-2A, or ZnT8A	9/22

**Table 3:** Distribution of serum antibody and specific-reactive T cells in 10 Chinese FT1D patients.

Patients	Serum antibody			Specific-reactive T cells		
	GADA	IA-2A	ZnT8A	GAD65	Insulin B9–23 peptide	C peptide
1	N	N	N	P	P	N
2	P	N	P	N	P	P
3	N	N	N	P	N	P
4	N	P	N	P	P	N
5	N	N	N	N	N	N
6	P	N	N	N	N	N
7	N	N	N	P	P	P
8	N	N	N	P	N	P
9	N	N	N	P	N	N
10	P	N	N	P	N	N

P: positive, N: negative.

**Table 4:** Distribution of GAD65 and insulin B9–23 peptide and C peptide reactive T cell in 10 Chinese FT1D patients.

Specific-reactive T cell	FT1DM (*n* = 10)
GAD65	7/10
Insulin B9–23 peptide	4/10
C peptide	4/10
GAD65 and insulin B9–23 peptide	3/10
GAD65 and C peptide	3/10
Insulin B9–23 peptide and C peptide	2/10
GAD, insulin B9–23 peptide, and C peptide	1/10
GAD, insulin B9–23 peptide, or C peptide	8/10
